# Allergen Microarrays and New Physical Approaches to More Sensitive and Specific Detection of Allergen-Specific Antibodies

**DOI:** 10.3390/bios14070353

**Published:** 2024-07-20

**Authors:** Pavel Sokolov, Irina Evsegneeva, Alexander Karaulov, Alyona Sukhanova, Igor Nabiev

**Affiliations:** 1 Life Improvement by Future Technologies (LIFT) Center, 143025 Moscow, Russia; 2 Laboratory of Nano-Bioengineering, National Research Nuclear University MEPhI (Moscow Engineering Physics Institute), 115409 Moscow, Russia; 3 Department of Clinical Immunology and Allergology, Institute of Molecular Medicine, Sechenov First Moscow State Medical University (Sechenov University), 119146 Moscow, Russia; ivevsegneeva@yandex.ru (I.E.); drkaraulov@mail.ru (A.K.); 4 Laboratoire BioSpecT, Université de Reims Champagne-Ardenne, 51100 Reims, France; alyona.sukhanova@univ-reims.fr

**Keywords:** allergen microarrays, sensitivity, specificity, antibody detection, quantum dots

## Abstract

The prevalence of allergic diseases has increased tremendously in recent decades, which can be attributed to growing exposure to environmental triggers, changes in dietary habits, comorbidity, and the increased use of medications. In this context, the multiplexed diagnosis of sensitization to various allergens and the monitoring of the effectiveness of treatments for allergic diseases become particularly urgent issues. The detection of allergen-specific antibodies, in particular, sIgE and sIgG, is a modern alternative to skin tests due to the safety and efficiency of this method. The use of allergen microarrays to detect tens to hundreds of allergen-specific antibodies in less than 0.1 mL of blood serum enables the transition to a deeply personalized approach in the diagnosis of these diseases while reducing the invasiveness and increasing the informativeness of analysis. This review discusses the technological approaches underlying the development of allergen microarrays and other protein microarrays, including the methods of selection of the microarray substrates and matrices for protein molecule immobilization, the obtainment of allergens, and the use of different types of optical labels for increasing the sensitivity and specificity of the detection of allergen-specific antibodies.

## 1. Introduction

Recent studies have clearly shown that allergic diseases are on the rise in both developed and developing countries, among not only children, but also adults [1]. Common examples of allergic diseases are food allergy and allergic asthma, hay fever, atopic dermatitis, and some others, their severity varying from minor manifestations to life-threatening reactions [2]. Allergic reactions develop in response to contact with relatively safe compounds as a result of the individual hypersensitivity of the patient’s immune system. Not only the number of people susceptible to allergies but also the number of newly identified compounds that cause allergic reactions are increasing each year. For example, within slightly more than three years, from January 2019 to March 2021, 106 new allergens, i.e., an average of 47 new allergens per year, were included into the WHO/IUIS Allergen Nomenclature Database, whereas an average of 33 new allergens per year were included between 2008 and 2018 [3]. The clinical relevance of most of these allergens is still to be confirmed, but the diagnostic approaches should be regularly updated to facilitate the unraveling of the clinical relevance of allergens and the development of personalized medicine. The diagnosis of allergic diseases relies on clinical history and laboratory tests. Sensitization, i.e., the presence of the corresponding allergen-specific IgE, IgG, and/or IgA antibodies (sAbs) [4], is confirmed either by skin tests or by in vitro allergen-specific IgE (sIgE) assays. Bignardi et al. performed a retrospective study of 793 patients and found that the results of tests for serum sIgE and skin tests agreed well, although their sensitivity and specificity varied for different allergens [5]. The diagnosis of allergic diseases is an ideal example of personalized medicine, because each patient has a unique allergic sensitization profile. However, effective diagnosis requires methods that allow for the simultaneous detection of multiple sAbs, and traditional skin tests and ELISA are unsuitable for this purpose because they are laborious, expensive, and time-consuming. Furthermore, skin tests have to be repeated dozens of times with different allergens, which excessively traumatizes the patient and may cause anaphylactic shock, though its risk is no higher than 0.02% [6,7,8]. In addition, it has been shown that detailed sAb profiling can increase the effectiveness of allergen-specific immunotherapy [9]. Cohort studies show that allergen microarrays are powerful tools not only for the diagnosis of allergy and for allergen immunotherapy stratification but also for assessing the future risk of allergy. Early sIgE reactivity to several allergen molecules has been found to be a predictive marker of respiratory allergy later in life [10]. That study used MeDALL microarrays with a conventional cut-off of 0.3 ISU for a positive sIgE response; however, a recent analysis has shown that a cut-off of 0.1 ISU provides better prediction and allows for the earlier detection of clinically relevant IgE sensitization. Allergen immunotherapy is known to be more successful at earlier stages of disease; therefore, the detection of low sIgE levels is important for a timely start of allergen immunotherapy, especially in pediatrics, which calls for allergen microarrays with enhanced sensitivity. The possibility of analyzing small volumes of biological fluids makes it possible to study not only blood serum samples, but also, e.g., tears, and is extremely relevant for pediatrics, where the blood sample volume is often limited. Research and clinical diagnosis use microarray technologies to detect multiple markers. There are DNA microarrays for detecting genetic markers, polymorphisms, and single nucleotide substitutions [11] and protein/antibody microarrays for detecting markers of autoimmune diseases [12], cancer [13], and many other diseases [14]. Allergen microarrays, such as ImmunoCAP™ ISAC™ (Thermo Fisher Scientific, Waltham, MA, USA) and Allergy Explorer (ALEX) (MacroArray Diagnostics GmbH, Vienna, Austria), are already successfully used in clinical practice to detect sIgE in serum samples. There are also about a dozen companies whose solid allergen microarrays have not yet been approved for clinical diagnosis but are used in research, e.g., OmicsArray™ Allergen Antigen Array (GeneCopoeia, Inc., Rockville, MD, USA), Allergen Epitope Microarray (PEPperPRINT GmbH, Heidelberg, Germany), Microtest (Microtest Matrices Ltd., London, UK), and IgE-QBA™ (Indoor Biotechnologies, Charlottesville, VA, USA). There are also suspension microarray techniques that employ routine flow cytometry [15] and ultrasensitive digital ELISA techniques with sub-attomolar detection limits [16]. Dozens of research groups annually publish hundreds of papers on the use of allergen microarrays and the development of new ones. The goal of this review is to systematize the approaches to the development of allergen microarrays and to summarize the current technologies of the fabrication of their elements that are already used or can be used in designing new, improved allergen microarrays. A special focus is made on nanostructured solid supports and different approaches to fluorescence signal amplification for higher specificity and sensitivity.

## 2. Structural Elements of Allergen Microarrays

**Figure 1:**
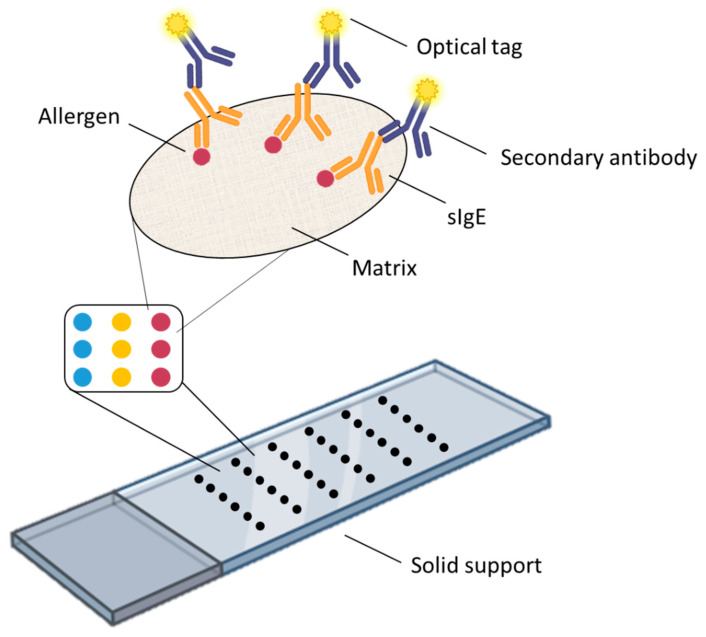
Schematic of an allergen microarray.

Although there are suspension allergen microarrays, such as IgE-QBA™, where the allergens are immobilized on the surface of microbeads and flow cytometers are used for analysis [15], most allergen microarrays are solid-state, with their typical structure shown in Figure 1. Basically, an allergen microarray consists of the following key elements: the microarray substrate, which can be made of glass, silicon, or other materials and usually has the same size as a standard microscope slide; a special matrix applied on the substrate surface to immobilize allergen molecules (hereinafter, by allergens we mean purified natural allergens, recombinant allergens, and allergen extracts); and the allergens themselves. For sIgE detection, a blood serum sample containing sIgE is applied onto the surface of the microarray. These sIgE antibodies selectively bind their respective antigens (allergens), thus being immobilized in a specific area of the microarray, after which they are detected using anti-sIgE antibodies conjugated to optical labels. The results of these allergen microarray assays are analyzed using a special reader or a fluorescence microscope. This is the most general schematic of the solid-state allergen microarray and sIgE detection principle; the specific design of the microarray may vary, but it always contains the above three elements or their analogs. Below, we will discuss each element of the microarray, as well as the approaches used for detecting sAbs, and analyze how a particular element affects the performance of allergen microarrays and what technologies are used in other microarrays and could be successfully applied to allergen microarrays.

## 3. Microarray Substrates

Traditionally, glass microscope slides, as well as silicon and polymer flat substrates, serve as the microarray substrates. The substrate is used not only for applying printing matrices and immobilizing allergens on it but sometimes, depending on the material and structure of the substrate, also for enhancing the fluorescence signal of the optical labels used for sAb detection. Solid flat substrates are more commonly used for microarray printing because they ensure the high-precision printing of small volumes of liquids. For example, NanoPrint™ (Arrayit Corporation, Sunnyvale, CA, USA) allows spots as small as 37.5 µm in diameter to be obtained upon application of less than one nanoliter of solution. Initially, the surface of glass slides has a negative electrical charge, which allows for the immobilization of protein molecules only through electrostatic interaction. This does not ensure reliable immobilization, because protein molecules can detach from the microarray surface, e.g., when the pH changes. To solve this problem, commercially available glass slides with active groups deposited on their surface, such as epoxy groups, amino groups, aldehyde and methacrylate groups, biotin, streptavidin, proteins A and G, and hydrophobic groups, are generally used. They will be discussed in more detail in the next section. There are also commercially available plastic substrates for microarray printing (produced, e.g., by PolyAn) that are preliminarily functionalized with carboxyl and amino groups, polylysine, streptavidin, N-hydroxysuccinimide (NHS) ester, or other compounds, which enables the efficient immobilization of proteins. In addition, these substrates are made of low-autofluorescence polymers to increase the signal-to-noise ratio [17]. Gerdtsson et al. [18] investigated microarray printing substrates made of different materials whose surface was functionalized for binding proteins through covalent bonding, electrostatic interaction, or adsorption. They concluded that, regardless of the type of surface coating, the most homogeneous background signal, as well as the highest reproducibility of spot size and shape upon printing, were obtained in tests with Black MaxiSorp polystyrene substrates. Piruska et al. [19] studied the autofluorescence characteristics of poly(methyl methacrylate) (PMMA), cyclic olefinic copolymer, polycarbonate, and poly(dimethylsiloxane) (PDMS) plastic microarray substrates and compared them with those of borosilicate glass substrate under laser irradiation at 403, 488, 680, and 780 nm. They found that borosilicate glass had the weakest autofluorescence at all wavelengths tested. All the polymeric materials had an autofluorescence peak at 403 nm, the PDMS and PMMA fluorescence rates being the lowest. The conclusion was that it is preferable to use optical labels excited at longer wavelengths in order to reduce the autofluorescence of the substrate.

Solid substrates can be used not only for the immobilization of allergens but also for increasing the sensitivity of sAb detection. Cretich et al. [20] analyzed the effect of fluorescence signal enhancement by a silicon oxide layer formed on the surface of a silicon substrate. They found that one of the simplest methods for enhancing the fluorescence was optical interference coating technology, using substrates with one or more layers of precisely specified thickness to increase the optical absorption of dye molecules near the surface and reflect emitted light, which would normally be lost in the substrate. The authors studied silicon oxide layers of different thicknesses on the surface of a silicon substrate and experimentally found that the 100 nm layer ensured the maximum enhancement of Cy3 dye fluorescence (by a factor of five to ten compared to the conventional glass substrate). Moreover, the signal-to-noise ratio of microarrays on the modified silicon substrates was four to seven times higher compared to those on glass substrates. Another study by Cretich et al. [21] showed a similar enhancement of the sensitivity of allergen microarrays on a substrate coated with a silicon oxide layer. The authors compared microarrays on a glass substrate and on a silicon substrate with an 80 nm reflective silicon oxide layer and found that the former could detect low concentrations of sIgE against Bet v 2 in only four of seven sera tested, whereas the latter could do so in all the seven sera. Multilayer substrates can also be used for enhancing the fluorescence signal from optical labels. For example, Petralia et al. [22] used a Si/Al/SiO_2_ substrate in which the fluorescence of the Cy5 dye was enhanced due to the reflection of optical radiation by a 900 nm Al film and due to the enhanced absorption of the excitation radiation by the dye placed at a given distance from the reflecting layer, which was ensured by a 830 nm SiO_2_ layer.

**Figure 2:**
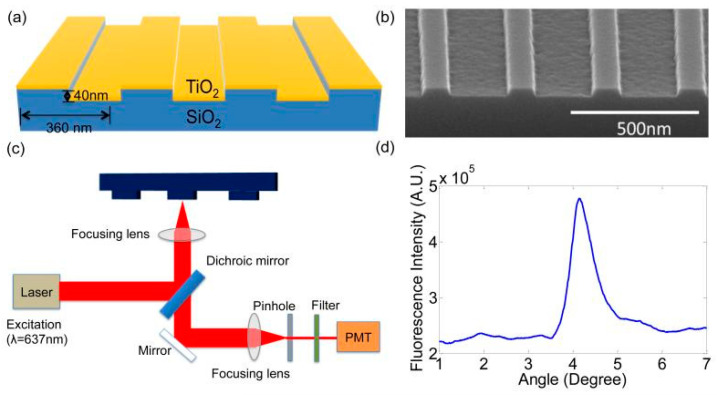
(**a**) A schematic of the photonic crystal (PC) structure and the laser scanning detection instrument. The PC is comprised of a periodic surface structure fabricated in a low-refractive-index (RI) silicon dioxide (SiO_2_) layer on a silicon substrate that is overcoated with a thin film of high-refractive-index TiO_2_. (**b**) An SEM image showing the surface structure of the PC. (**c**) A schematic diagram of the detection instrument. (**d**) Reflected intensity as a function of incident angle of the PC when it is illuminated by a transverse magnetic (TM) polarized laser at a wavelength of λ = 637 nm. The peak location of the spectrum indicates the resonance condition is achieved at the incident angle of 4.12. Reprinted with permission from Tan et al. [23]. Copyright 2015 Elsevier.

There are other examples of increasing the sensitivity of fluorescent signal detection by modifying the surface of substrates. For example, Tan et al. [23] used a substrate containing a photonic crystal (PC) to enhance fluorescence in detecting sIgE against the Fel d 1 allergen. The PC consisted of a periodic surface structure fabricated on a silicon substrate by photolithography and the reactive ion etching of a SiO_2_ grating structure (period, 360 nm; grating depth, 40 nm), which was coated with a thin film of TiO_2_ (thickness, 130 nm). After this, the wafers were diced into 1.0 × 0.5 in pieces. A single 0.5 × 1.0 in PC die held 10 subarrays, each containing 4 sets of 4 replicate spots per protein, i.e., a total of 16 spots. Before allergen printing, the PC surface was activated using epoxysilane chemistry to ensure a high binding capacity for capture antibodies. Microarrays were printed by means of a commercially available spot printing system using standard equipment without optimization for printing on a PC surface. The measured spot diameters were 79.00 ± 2.22 µm, row spacing was 149.25 ± 3.26 µm, and column spacing was 200.75 ± 0.82 µm. The detection sensitivity was increased due to two independent effects exerted by the photonic crystal: an increase in excitation and an increase in extraction. The increase in excitation was achieved by using a periodic nanostructure that acted as an optical resonator operating at the wavelength of the excitation radiation. An enhanced electric field related to standing electromagnetic waves was formed in the evanescent field region at a distance of about 100–200 nm from the photonic crystal surface. As a result, fluorescent labels bound to the photonic crystal surface were exposed to stronger excitation radiation. Extraction was enhanced by creating a photonic crystal surface that also provided a second optical resonance at the emission wavelength of the fluorescent label, which allowed for the preferential extraction of emitted photons in the direction perpendicular to the photonic crystal plane, thereby increasing the photon collection efficiency. The structure of the substrate that was used and the schematic detection setup are shown in Figure 2. As a result, the authors succeeded in detecting sIgE at a concentration of 0.02 kU/L (approximately 0.048 ng/mL or 0.035 ISAC standardized units (ISU), which is three to ten times smaller than the amounts detected by standard FDA-approved tests. For example, the cut-offs for ImmunoCAP and MeDALL in those tests were as high as 0.3 and 0.1 ISU, respectively [24,25].

In addition to the one-dimensional photonic crystals described above, two-dimensional photonic crystals consisting of arrays of nanorods (also made of semiconductors) can be formed on the surface of substrates. For example, Verardo et al. [26] fabricated a substrate containing a two-dimensional photonic crystal consisting of GaP nanorods coated with a 10 nm layer of SiO_2_ that enhanced the fluorescence of Alexa Fluor 647 dye. The nanorods were 4 µm in height and about 160 nm in diameter and were located about 3 µm away from one another. The detection sensitivity was increased because this structure increased the surface area compared to planar substrates, with the nanorods collecting the emission from a larger number of fluorophores bound to the surface and re-emitting it at the tip, similarly to optical fibers [27], thereby significantly increasing the total emission intensity to allow for the detection of even single molecules [28]. Second, as in the aforementioned one-dimensional photonic crystal, the excitation and extraction of the fluorescence signal from the optical labels were enhanced. The authors used this approach for the detection of serum biomarkers recognized by the ScFv antibody fragments immobilized on the microarray; however, it is also suitable for the detection of sAbs.

Surface plasmon coupled emission (SPCE), which is determined by the interaction between surface plasmons and excited fluorophores, is another example of surface-enhanced fluorescence. This process can be considered the inverse of surface plasmon resonance: if the incident light and surface plasmon satisfy the condition of wave vector matching, the incident light is absorbed. SPCE is generated by the interaction between excited fluorophores in the near field of a metal substrate and surface plasmons [29]. Yuk et al. [30] described a similar substrate in the form of a plate of ZEONEX^®^ (Zeon corporation, Tokyo, Japan) cyclic olefinic polymer on whose surface a 3 nm layer of chromium and a 50 nm layer of gold were deposited. They demonstrated that this microarray could be used in combination with antibodies fluorescently labeled with Alexa Fluor 647 to detect human IgG with a detection limit of 10 ng/mL.

It should also be noted that the accuracy and sensitivity of the measurement of sAb concentration in serum depends on the reproducibility of allergen printing on the microchip surface. Monroe et al. [31] proposed a method of calibrated fluorescence enhancement (CaFE), which allows for not only increasing the sensitivity of sIgE detection by enhancing the fluorescence of the optical labels but also calibrating the results using label-free estimation of the number of allergens printed on the microarray surface. For this purpose, the authors used interferometric reflectance imaging sensor (IRIS) technology [32]. The microarray consisted of two areas. In one area, a 100 nm silicon oxide layer was formed on the silicon substrate surface to enhance the visible-range fluorescence of the dyes typically used for detection; in the other area, the thickness of the silicon oxide layer was 500 nm, which was optimal for label-free detection by the IRIS method. This substrate made it possible to increase the fluorescence intensity of the Cy3 dye by a factor of two compared to the conventional glass substrate. Unfortunately, the results of fluorescence signal detection using two different batches of microarrays poorly agreed with each other (with R^2^ ranging from 0.24 for the Phl p 1 allergen to 0.88 for the Ara h 1 allergen) due to the different physicochemical properties of the allergens, such as the affinity of immobilization on the surface, as well as technical aspects of microarray manufacturing, such as printing irregularity. However, normalization by means of label-free determination of the amount of printed allergen increased the agreement between the results to R^2^ = 0.914 for Ara h 1 and R^2^ = 0.9 for Phl p 1.

Thus, the allergen microarray substrate not only serves for the immobilization of allergens but also can amplify the fluorescence signal from detection labels. The most popular substrate for microarrays is a glass slide. Table 1 shows a comparison of different substrates with glass-based substrates. There is no doubt that modern substrates are more sensitive in analyte detection due to a higher signal-to-noise ratio and fluorescence enhancement, but they are more expensive and less suitable for scalable and reproducible production than glass slides. The effective surface area and capacity of protein immobilization, as well as spot shape and size reproducibility, are more dependent on the matrices used for immobilization, due to different porosity and wetting properties [18]. On the other hand, since the characteristic dimensions of the roughness of photonic crystals are usually no more than 100 nm in height, whereas the spot size during printing is about 1–100 μm and printing occurs on a matrix-treated surface, the protocols for printing on glass substrates and nanostructured substrates are the same [33,34,35].

## 4. Matrices and Methods for Allergen Immobilization

Various approaches are used to immobilize allergens on the substrate, including covalent bonding, 2D adsorption, the diffusion of molecules into a thick matrix (3D adsorption), and affinity interactions. Physical adsorption is the simplest method of allergen immobilization because it does not require additional functionalization of the substrate surface, the immobilization being due to electrostatic interaction and van der Waals force. A disadvantage of this approach is that the interaction is weak and can be destroyed by changes in pH, the ionic strength of the solvents used in the analysis, or even temperature. This type of immobilization was used, e.g., by Tortajada-Genaro et al. [37] in printing allergens onto the surface of a polycarbonate DVD disc. Before printing, the disk did not undergo any special treatment, but was only washed with ethanol and water, and allergens dissolved in 50 mM carbonate buffer solution (pH 9.6) containing 1 vol% of glycerol were used for printing. The resultant microarray allowed for the simultaneous detection of up to 12 sIgE antibodies against food allergens in a serum sample as small as 25 μL with a detection limit of down to 0.3 IU/mL. Physical adsorption on polyvinylidene difluoride (PVDF) or nitrocellulose (NC) membranes can also be used. Glass substrates coated with PVDF or NC layers are commercially available, e.g., from Arrayit and Grace Bio-labs [38]. PVDF coating is less commonly used for allergen microarrays than NC, because NC more readily binds low-molecular-weight proteins [39]. However, the use of PVDF and NC coatings in microarrays is often limited because of the high level of nonspecific binding and the resulting high background signal [40].

Hydrogels are used for increasing the allergen-binding capacity of microarrays. Advantages of this approach are the better preservation of allergen conformation and their spatial arrangement in the 3D matrix, which increases the efficiency of sIgE binding to immobilized allergens [41,42]. For example, Feyzkhanova et al. [43] used a mixture of 3.7% methacrylamide (*w*/*v*), 0.3% N,N′-methylene bis-acrylamide (*w*/*v*), and 50% glycerol (*v*/*v*) containing allergens with an attached methacryl group to obtain a hydrogel with allergens immobilized in it. After the microarray was printed onto a glass substrate, it was irradiated with UV light for polymerization. This allergen microarray allowed for the highly sensitive detection of sIgE antibodies against 21 allergens.

Covalent immobilization requires the functionalization of the microarray substrate surface with various compounds that specifically bind NH_2_, COOH, or OH groups of the protein molecules of allergens. For example, treatment of the substrate with N-hydroxysuccinimide (NHS) ester [44], aldehydes [45], and isocyanate [46] allows for the immobilization of allergens via NH_2_ groups. Kalli et al. [45] compared microarray coatings consisting of aldehyde, epoxy, and NHS esters as agents facilitating the immobilization of κ-casein, timothy grass pollen extract proteins, and polyclonal antibodies against human IgE. The authors found that NHS ester was the best at preserving the original physiological activity of the immobilized proteins. Jeon et al. [44] used glass substrates functionalized with a hydrophilic polymer and NHS ester to immobilize allergens. NHS ester functionalization ensured the irreversible and stable immobilization of allergens, and the hydrophilic polymer coating prevented the nonspecific adsorption of random proteins and minimized the background signal. The microarray designed in that study allowed quantitative data to be obtained in a linear sIgE concentration range from 0.35 to 100 IU/mL. Suzuki et al. [47] described the design of an allergen microarray with a high density of immobilized allergen molecules for the highly sensitive detection of sIgE. A high immobilization density was ensured by using carboxylated arms on the surface of a diamond-like carbon (DLC) microarray. To activate the carboxyl groups of the DLC film, the microarray was incubated in the presence of freshly prepared 100 mM 1-ethyl-3-(3-dimethylaminopropyl) carbodiimide (EDC) hydrochloride and 20 mM NHS. The aldehyde groups on the surface of the glass substrate reacted with primary amines of the protein to form bonds via Schiff bases. Measurements in serial dilutions of serum samples showed that the sensitivity of sIgE detection was increased by a factor of four to eight compared to the commercially available UniCAP kit (Phadia, Uppsala, Sweden). Maleimide treatment of the microarray substrate surface allows for the covalent immobilization of proteins via SH groups [48]. However, it should be noted that only two proteinogenic amino acids, cysteine and methionine, contain SH groups, and if the residues of these amino acids are important for the functional activity of the allergen, then immobilization via these groups leads to the loss of functional activity of the allergen.

The background signal or noise due to the nonspecific binding of biomolecules to the activated microarray substrate is one of the most common problems limiting the sensitivity of analysis. The intensity of the background signal is usually higher if complex biological fluids are analyzed, because they contain a combination of molecules and vesicles that may adsorb on the substrate surface. Ströhle and Li [49] analyzed strategies of blocking nonspecific binding using bovine serum albumin, skim milk, polyethylene glycol, and a protein-free blocker. They showed that blockage with proteins yielded the best results in analyzing serum and plasma samples if the substrate surface was functionalized with nitrocellulose, whereas the protein-free blocker was the best in the case of extracellular vesicle lysates if the substrate surface was functionalized with 3-glycidoxypropyltrimethoxysilane and nitrocellulose. Thus, the selection of the optimal blocking agent depends not only on the type of surface functionalization but also on the type of the analyzed sample.

Cretich et al. [21] used a functional DMA–NAS–MAPS copolymer for allergen immobilization. This copolymer formed a nanometer-thin layer on the substrates, which made it possible to completely preserve the topography of the substrate surface and not to disturb its optical properties, which is important when nanostructured substrates are used. The main chain consisted of a DMA monomer forming hydrogen bonds with the silicon surface, which resulted in a hydrophilic interface capable of preventing nonspecific protein adhesion. The functionalized NAS monomer bound protein molecules, whereas MAPS monomers contributed to film stability by reacting with surface silanes.

In addition, the spatially oriented immobilization of protein molecules can be included in the printing process by using affinity tags, such as biotin–streptavidin [50], glutathione-S-transferase (GST)–anti-GST antibody [51], or His-tag–Ni^2+^-NTA [52] pairs. This allows the structure of immobilized allergens to be preserved by immobilizing them via a tag located at the N or C end of the protein molecule. This method is usually used to fabricate protein microarrays by means of the in situ expression of proteins from immobilized DNA matrices [52]. This technology can reduce the cost of microarray production, because chemical synthesis of DNA is faster and less expensive than the chemical synthesis of proteins or their bacterial expression and purification. Li et al. [50] fabricated a microarray of this type using a substrate functionalized with streptavidin and special plasmids expressing biotinylated recombinant proteins. After in vitro expression and translation, the proteins were immobilized on the microarray surface through biotin–streptavidin interaction. However, this method does not allow for precise control of the amount of immobilized recombinant proteins and cannot yet be used to fabricate microarrays for quantitative analyses because of the low reproducibility of the microarray production process.

Ohyama et al. [53] developed an allergen microarray where allergens were immobilized using a photoreactive crosslinker. For this purpose, they coated polystyrene substrates with a polymer matrix containing polyethylene glycol (PEG-350) in the side chains. For printing, they used allergens mixed with the disodium salt of 4,4′-diazidostilbene-2,2′-disulfonic acid serving as a crosslinker. After printing, the microarray was irradiated with UV light, which caused allergens to bind to PEG-350. This method is versatile in that it allows for the immobilization of any recombinant protein regardless of the functional chemical groups on its surface.

A unique allergen immobilization technique is used in ALEX microarrays (MacroArray Diagnostics GmbH). Allergens are initially bound to activated nanoparticles, which optimizes the binding conditions for each individual allergen. The immobilization of each allergen is tailored to its biochemical properties and stability requirements, thereby completely preserving the functionality of the epitope to which the sAbs bind. The nanoparticles increase the surface area of the solid phase, which ensures complete allergen exposure during the immunoassay and, hence, highly sensitive detection of sAbs. At the next step, allergen-bearing nanoparticles are deposited onto the solid matrix, forming a macroscopic array of individual allergens for analysis [54].

To conclude this section, the study by Guilleaume et al. [55] is worth mentioning. They compared combinations of different methods of protein immobilization on microarrays and fluorescent detection labels. In particular, they found that the reproducibility and accuracy of the results obtained using different fluorescent dyes of the Alexa Fluor family varied depending on the matrix and the protein immobilization method, which indicated the need for the careful selection of matrices and allergen immobilization methods depending on the fluorescent label planned for detection. The main methods of protein immobilization and matrices used for this purpose are shown in Table 2.

## 5. Allergens Used in Microarrays

The functioning of allergen microarrays requires that, after the specified number of allergens have been printed on the microarray surface, they could be specifically bound by the corresponding sAbs. Both recombinant allergens and native allergens purified from natural sources and allergen extracts can be used for printing [56]. Here, recombinant and purified allergens are more preferable, because allergen extracts may contain glycoproteins and other impurities that may bind nonspecifically with sAbs. The epitopes of allergens derived from plants and insects contain α-1,3-fucose on an asparagine-linked N-glycan residue. N-glycans carrying this epitope are widely distributed; they are collectively known as cross-reactive carbohydrate determinant (CCD), which is often a source of false-positive results in the detection of sIgE and other allergen-specific antibodies when allergen extracts are used [57]. In addition, the concentration of allergen in the extract may vary from batch to batch, which makes it difficult to control the amount of immobilized allergens on the microarray surface and may cause inaccuracies in the determination of sAb concentrations. Buzzulini et al. [58] compared the data obtained using the ALEX allergen microarray, which contains both allergen extracts and recombinant allergens, with those obtained using the ImmunoCAP ISAC, which does not contain allergen extracts. A qualitative comparison of sIgE detection showed agreement between the data obtained using ALEX and ImmunoCAP; however, in the case of allergen extracts and pure allergens, the agreement, as estimated by the Cohen’s kappa coefficient, was k = 0.64 for inhalation preparations and k = 0.51 for food allergens, whereas in the case of pure allergens, the agreement was considerably higher: k = 0.92 and k = 0.72, respectively. Nevertheless, a quantitative comparison showed a low correlation between the results obtained using ALEX and ImmunoCAP.

Purified allergens and recombinant allergens are much more amenable to standardization and are more commonly used in diagnostic tests because they allow researchers to obtain more accurate and specific information than allergen extracts. There are many techniques for obtaining recombinant allergens. The most common systems for the expression of recombinant proteins are microbial expression systems, mainly based on different strains of *E. coli* [59] and yeast [60]. This is due to both well-developed molecular genetic methods for the cloning and translation of genetic information and the simple bacterial culture procedure. For example, Wallner et al. [61] described methods for laboratory and semi-industrial production of the recombinant allergen Bet v 1 and discussed the selection of optimal vectors for expression, conditions for cell induction and culturing, etc. Neophytou et al. [62] reported a detailed protocol for the production of the recombinant Pru p 3 allergen from peach in *P. pastoris* cells. There are also examples of producing recombinant allergens in plant and mammalian cells [63,64]. It should be noted that the use of eukaryotic cells for allergen expression is more preferable, because they are capable of post-translational modification (PTM), protein processing, and more correct folding than bacterial cells, which makes it possible to obtain allergens with an activity close to that of native allergens. About half of the proteinogenic amino acids can be modified, with these modifications changing the molecular weight of the protein, e.g., by 14, 42, 80, and 2–3 Da in the cases of methylation [65], acetylation [66], phosphorylation [67], and the addition of oligosaccharide structures, respectively. Amino acids whose side chains contain hydroxyl, amino, or thiol groups, such as serine, threonine, tyrosine, histidine, aspartate, aspartagine, lysine, arginine, and cysteine, and, hence, allergens containing the residues of these amino acids, are the most prone to PTM. The PTM of the allergen polypeptide chain can affect the specificity of its binding with the sAb. Protein folding or the formation of the tertiary protein structure, i.e., its native spatial structure, occurs during the synthesis of the polypeptide chain [68]. Proteins that have not acquired the correct structure during translation usually precipitate in the form of inclusion bodies and lack functional activity. Because folding of the polypeptide chain occurs simultaneously with translation, a decrease in the translation rate leads to an increase in the solubility of the protein and its more correct conformation. This is usually accomplished by lowering the bacterial culture temperature at which induction occurs, reducing the amount of the transcription inducer [69], or adding glucose in order to catabolically reduce the rate of protein expression [70]. It is also known that induction of heat shock proteins, including chaperones, improves protein folding and reduces the formation of inclusion bodies [71]. Two methods not requiring expensive equipment are most commonly used for monitoring protein folding. The first method is thermofluorescence, which is based on the detection of conformational changes in the protein molecule upon heating [72]. For this purpose, fluorescent dyes are used, e.g., SYPRO Orange, which binds nonspecifically with hydrophobic parts of protein molecules, whereas water strongly quenches its fluorescence. The protein globule contains hydrophilic residues on its surface, all hydrophobic sites being inside the globule, which allow the protein to remain in solution. When the temperature is raised, the normally coiled protein globule begins to unfold, SYPRO Orange binds with the hydrophobic sites, and its fluorescence is restored. The proteins that have folded incompletely or incorrectly during synthesis already have hydrophobic sites on their surface; hence, the SYPRO Orange fluorescence signal can be detected. The second method makes use of the differences in the absorption of circularly polarized light between different protein conformations, which makes it possible to employ the phenomenon of circular dichroism [73]. α-helices and β-sheets are the most common secondary structures of all protein molecules, and their circular dichroism spectra have characteristic shapes. Therefore, by varying the physicochemical parameters of the protein solution and measuring the circular dichroism spectra, a researcher can judge whether the protein folding is correct.

Recombinant allergens are usually purified using affinity chromatography. For this purpose, a nucleotide sequence encoding an affinity tag is inserted into the nucleotide sequence of the expressed allergen at the 3′ or 5′ end. In this case, a translational fusion of the affinity tag with the allergen at its N- or C-terminus is obtained after the expression. Affinity tags can be short peptides. For example, five arginine residues allow for the efficient purification of proteins by cation-exchange chromatography, six histidine residues are suitable for purification by metal-affinity chromatography, and an oligopeptide tag with the amino acid sequence DYKDDDDDDK (FLAG) allows for the the purification of proteins using immobilized antibodies against FLAG. The use of short amino acid tags minimizes interference with the folding of short proteins and polypeptides [74]. In addition, proteins that increase the solubility of recombinant proteins, such as maltose-binding protein (MBP), thioredoxin (Trx), and ubiquitin-like modifier (SUMO) proteins, can be translationally attached to recombinant proteins to enable affinity purification [75]. The short peptide or protein tags can be cut off from the recombinant protein after purification, for which purpose a protease site can be inserted between the recombinant protein and the tag. The proteases most commonly used for this purpose are the tobacco etch virus (TEV) protease [76], thrombin, factor Xa protease, and some others [77]. The purification of native allergens from extracts is usually more laborious and technically more complicated. For the purification of native allergens from extracts, sequential combinations of different types of chromatography are used, including gel-filtration chromatography, which separates molecules in solution according to their size [78]; ion-exchange chromatography, which is based on the reversible binding of charged molecules on the active groups of the resin [78]; reverse-phase (hydrophobic) chromatography, which separates molecules according to their hydrophobicity [79]; and affinity chromatography, which separates proteins according to the reversible interaction between a protein (or a group of proteins) with a specific ligand linked to the chromatographic matrix. Affinity chromatography using antibodies immobilized on the stationary phase that selectively bind a specific allergen is the most effective method for obtaining pure native allergens; however, the spectrum of such antibodies is small; hence, the use of this purification method is limited [80].

Current biotechnological methods have made the obtainment of recombinant proteins easy, inexpensive, and quite fast; however, there is no unequivocal answer as to which types of allergens are more suitable for printing as components of microarrays, because each of them has its own advantages and drawbacks (Table 3).

## 6. Optical Labels for the Detection of Allergen-Specific Antibodies

Typically, secondary antibodies (usually IgG antibodies against sIgE) conjugated to organic fluorescent dyes are used to detect sIgE bound to allergens immobilized on the surface of the microarray. Thus, the specificity and sensitivity of detection are determined by the specificity and affinity/avidity of the antibodies against sIgE and the optical characteristics of the fluorescent labels. Not only secondary antibodies, but also, e.g., FcεRI, a receptor that binds the Fc site of sIgE [81], as well as fragments of full-length secondary antibodies or single-domain antibodies capable of binding sAbs, can be used for their detection. However, only full-length secondary anti-sIgE antibodies are currently used in microarrays. Here, we will not elaborate on their selection.

Optical labels used for the detection of sAbs should provide an intense optical signal that could be detected with a high sensitivity. Traditionally, the immunochromatographic assay (ICA) and enzyme-linked immunosorbent assay (ELISA) use colorimetric labels, which are less sensitive than fluorescent ones [82]. In microarrays, where the amount of immobilized allergens is smaller than in ELISA and ICA and, hence, less sIgE is bound, detection sensitivity is a particularly urgent issue. Some allergen microarrays also use colorimetric detection. For example, Lebrun et al. [83] used antibodies against human IgE conjugated to alkaline phosphatase, which converts 5-bromo-4-chloro-3-indolyl phosphate/nitro blue tetrazolium into an insoluble colored product. Tortajada-Genaro [37] described microarrays that used conjugates of antibodies against human IgE with horseradish peroxidase, which oxidizes tetramethylbenzidine, thus changing its color. However, fluorescence detection is the most common in microarray technologies and is used for detecting both allergens [84] and other analytes [13]. Fluorescence detection ensures high sensitivity and speed of detection, and fluorescent labels and detection equipment are inexpensive and commonly available. Radioactive labels could be an alternative to fluorescent ones because they provide even higher sensitivity, but they are potentially hazardous to health, and equipment for their analysis is less commonly available and more expensive than that for fluorescence detection.

Organic fluorescent dyes, such as the cyanine dyes Cy3 and Cy5 [85], as well as dyes of the Alexa Fluor [86] and DyLight [87] families, are the most commonly used fluorescent labels because they appeared on the market earlier. The best known and most mass-produced allergen microarray on the market is the ImmunoCAP ISAC. This microarray contains 112 allergens from 48 sources printed in triplets. Depending on the version of the allergen microarray, it contains antibodies against human IgE conjugated either to an organic fluorescent dye [88] or, in earlier versions, to β-galactosidase [89]. In the latter case, the fluorescence signal is generated by 4-methylumbelliferyl-β-galactoside, which is converted to a fluorescent state after β-galactosidase is cleaved from it. The allergen microarray developed in the MeDALL project contains more than 170 allergens and employs a detection principle similar to that of the ImmunoCAP ISAC, using a fluorescently labeled antibody against human IgE [90]. In contrast, another popular allergen microarray, ALEX, does not use fluorescent dyes for detection. Here, antibodies against human IgE are conjugated to an enzyme that converts a special substrate into an insoluble colored form. Accordingly, the amount of colorimetric dye localized near the allergen is proportional to the amount of sIgE in the sample. It should be noted that, unlike the ImmunoCAP ISAC, which yields only semi-quantitative data, the ALEX microarrays allow for quantitative analysis, although not over the entire range of sIgE concentrations [91].

Sometimes, the primary labeling of the sample with biotin followed by its detection by means of a fluorescent dye conjugated with streptavidin is used for analyte detection instead of fluorescently labeled antibodies. For example, El-Haibi et al. [92] investigated the regulation of signaling pathways in prostate cancer using Fullmoon Biosystems Inc. antibody microarrays. They labeled protein extracts from cells with biotin and then used the streptavidin–Cy3 conjugate for detection. Note that the use of a single versatile affinity tag conjugated with a fluorescent dye reduces the cost of the experiment, but it does not eliminate the problems related to the nonspecific biotinylation of proteins in the sample. In addition, this approach is rarely used in allergen microarrays because a single conjugate of an anti-IgE antibody with a fluorescent label is enough for the detection of all different sIgE. Furthermore, primary labeling of molecules in the sample may lead to false-positive and false-negative results due to variable labeling efficiencies for different epitopes of proteins, which may cause the inactivation of their binding sites [93].

The use of fluorescent labels allows for the detection of different classes of sAbs. For example, Feyzkhanova et al. [94] developed a microarray that detected not only sIgE, but also sIgG4, evaluating the effectiveness of anti-allergy therapy. The microarray contained two types of detection antibodies conjugated with different fluorescent dyes, anti-IgE–Cy5 and anti-IgG4–Cy3, with the fluorescence signals from the two dyes detected sequentially. Dottorini et al. [95] detected sIgE by means of mouse anti-human IgE antibodies, which, in turn, were detected using antibodies against mouse IgG conjugated to horseradish peroxidase (HRP). Tyramide-Alexa 555 served as a dye for detection, its interaction with HRP resulting in a transfer of the fluorescent label to the surrounding proteins. This approach allowed for increasing the number of fluorescent labels per IgE molecule, which enhanced the sensitivity of detection by one to two orders of magnitude due to multiple labeling with Alexa 555 compared to labeling Alexa 555 conjugated with anti-IgE antibodies. The same principle of fluorescent signal amplification was used by Williams et al. [96]. These studies indicate that there is a need for more sensitive optical labels.

Fluorescent quantum dots (QDs) are increasingly used for biological imaging as an alternative to organic fluorescent dyes [97]. Fluorescent QDs are semiconductor crystals several nanometers to several tens of nanometers in size, with their optical properties, such as fluorescence and absorption spectra, depending on their size, structure, and composition. The cores of the nanocrystals usually consist of elements of groups III–V, II–VI, or IV–VI of the periodic system and are often coated with an outer epitaxial inorganic shell, which improves the optical properties and makes the QDs more resistant to environmental factors [98]. The sensitivity of fluorescence detection is determined not only by the brightness of the label fluorescence itself, but also by the ratio between the levels of the useful fluorescence signal and the background autofluorescence from the sample molecules, by the matrix used for immobilization, and by the substrate material. The fluorescence of QDs is, on average, ten times brighter than that of organic dyes [99], which makes it possible to detect a fluorescent signal of about the same intensity from a tenfold smaller number of labels and, hence, to increase the sensitivity of detection by an order of magnitude. In addition, the autofluorescence signal can be weakened, with the detection sensitivity increasing accordingly, by forcibly separating the moments of excitation and the detection of the fluorescent signal in time. It is known that QDs have a longer fluorescence lifetime than organic dyes [100,101] and biological molecules, which makes it possible to detect the fluorescence signal from QDs when the autofluorescence signal level has already decreased below a specified level. For example, Giraud et al. [102] used this approach to study DNA microarrays, which allowed them to increase the signal-to-noise ratio by a factor of 1.8 and achieve a femtomolar sensitivity of analyte detection. In addition, QDs are more photostable; i.e., they retain their fluorescence properties under prolonged exposure to excitation radiation. Monton et al. [103] showed that the excitation of an Alexa Fluor organic dye and QDs with a laser at a wavelength of 561 nm at the maximum power reduced the brightness of the fluorescence of the organic dye by a factor of 5 within 55 s, while the brightness of the QD fluorescence was decreased by as little as 5% within 90 s and to 80–90% of the initial level within 5 min. This stability allows the QD fluorescence signal to accumulate over time, which also increases the detection sensitivity and signal-to-noise ratio. Another advantage of high photostability is the considerably easier handling of QD fluorescent labels, because there is no risk of photobleaching during the preparation for analysis; hence, incubation procedures under daylight or artificial light are simplified. In order to make QDs stable in biological fluids and aqueous solutions, their surface is usually functionalized with hydrophilic ligands [104], or they are encapsulated into polymer shells [105] to protect them from degradation and aggregation. QDs are currently used in different types of biosensors and diagnostic tests, including hydrogel-based sensors [106], ELSA [107], lateral flow tests [108], and tumor imaging [109], as well as for photodynamic cancer therapy [110]. The use of fluorescent labels based on QDs makes it possible to obtain weaker background signals, because organic fluorescent dyes, e.g., AlexaFluor, apparently interact with proteins in a nonspecific manner due to their hydrophobicity [111]. Experiments with an oligonucleotide microarray [112] have shown that the fluorescence intensity of the spots is logarithmically linear to the model miRNA from 156 to 20,000 pM, and the dynamic range is about two orders of magnitude, with a detection limit of about 0.4 fmol, which implies that QDs can be used to quantify analytes in a broad concentration range with high sensitivity.

Although QD fluorescent labels are relatively new, they have already been used in various types of microarrays. For example, Zhou et al. [113] described a microarray containing 82 different antibodies for studying colorectal cancer. In this microarray, cells from cancer cell lines and clinical samples were bound by immobilized antibodies, and imaging was performed using antibodies labeled with QDs (CD325-QD705, CD3-QD800, and CD3-QD605) or organic fluorescent dyes (CD326-AlexaFluor647 and CD3-Phycoerythrin). In that study, imaging with organic fluorescent dyes yielded more reproducible results than imaging with QDs. This was explained by an inefficient excitation of QD fluorescence by the source used in the microarray scanner, a decrease in antibody affinity during QD–antibody conjugation by the technique used in the study, and a suboptimal QD-to-antibody ratio in the resultant conjugates. Lafont et al. [114] studied the activity of DNA kinases using a home-made antibody microarray printed on glass substrates with QD-based fluorescence detection. Preliminarily biotinylated antibodies were detected using streptavidin conjugated to QDs. The authors found that the use of QDs instead of organic fluorescent dyes increased the detection specificity, because organic dyes, being hydrophobic, bound nonspecifically with the analyte molecules and the microarray surface. Although QDs are not used in allergen microarrays yet, they constitute a promising alternative to organic fluorescent dyes due to their outstanding optical properties. In addition, there are detection methods that do not require the binding of an additional affinity tag, labeled with a fluorescent dye or a radioisotope, to the analyte. Such methods are called label-free ones because the detection of analytes occurs on the basis of their binding in a given area of the microarray with one affinity label, with direct detection of the binding event. The binding event can be detected by a change in the reflection or propagation of an electromagnetic wave, a change in molecular weight, etc. Some common label-free detection methods, with a brief description of their advantages and drawbacks, are listed in Table 4. The main advantage of label-free detection is that only one affinity recognition molecule is required, which provides immobilization of the analyte in a specific area of the microarray. This significantly simplifies multiplexed detection and improves discovering new biomarkers, because a second recognition molecule conjugated with the detection tag is not required. Label-free detection methods also have other advantages over traditional label-based methods, including easier operation and a high throughput due to automated measurement. On the other hand, their main disadvantage is the high cost of equipment (e.g., for mass spectrometry and surface plasmon resonance-based methods). However, some of these methods (e.g., those based on polarization and interferometric measurement) are comparatively low-cost, which facilitates their use in point-of-care diagnosis.

**Figure 3:**
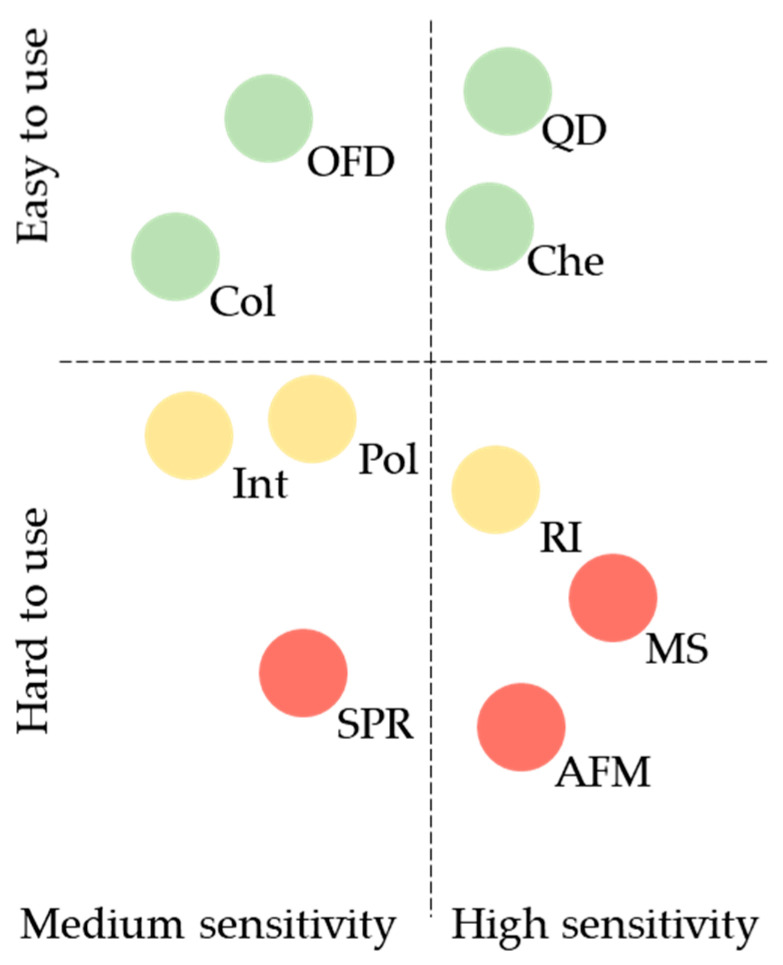
Essential criteria for designing diagnostic microarray sensors. QD, quantum dot-based detection; OFD, organic fluorescent dye-based detection; Che, chemiluminescence-based detection; Col, colorimetry-based detection; Int, interference-based detection; Pol, polarization-based detection; RI, radioisotope-based detection; MS, mass spectrometry-based detection; SPR, surface plasmon resonance-based detection; AFM, atomic force microscopy-based detection. Green circle, low cost; yellow circle, medium cost; red circle, high cost.

A comparison of label-free and label-based detection techniques regarding their sensitivity, ease of use, and cost, which are essential for designing diagnostic microarray sensors, is shown in Figure 3.

## 7. Conclusions

Allergen microarrays are a unique example of a diagnostic microarray technology, being significantly simpler than any other protein or antibody microarray. The assembly of an immune complex on their surface requires only allergens immobilized on the surface, a test sample containing sAbs, and fluorescently labeled antibodies against human antibodies. In contrast, antibody microarrays require antibody pairs that bind two different epitopes of the tested molecules. This makes allergen microarrays a versatile model for assessing and improving individual elements of solid-state microarrays. The main elements which can be improved in solid-state microarrays are the following:

– Solid substrates with nanoengineered surfaces (such as PCs or thin reflective layers for signal amplification) are characterized by minimal autofluorescence and simple, reproducible functionalization procedures. It is also necessary to further develop the technology for producing these nanostructured substrates to reduce the cost of their production and obtain substrates with reproducible structural and other characteristics.
– Matrices for the functionalization of the solid substrate surface should provide permanent immobilization of a sufficient amount of allergens, have a low autofluorescence level, and minimally affect the optical properties and structure of the solid substrate. The main problem is that, due to the different physicochemical properties of the allergens, such as the affinity for immobilization on the surface, the amounts of allergens immobilized on the substrate may vary significantly on different microarrays, and an ideal matrix should have the same immobilization specificity for different allergens.
– Detection can be performed by label-free or label-based techniques. The most popular currently available commercial products are microarrays with fluorescent labels due to their high sensitivity, large dynamic range, and comparatively inexpensive equipment for detection. The most promising techniques combine label-free detection for standardization of the amount of immobilized allergens and the use of optical tags for sIgE detection.

Glass printing technology is well established in clinical and research practice and is commercially successful. However, it is possible to further enhance measurement sensitivity and, hence, detect smaller amounts of antibodies in samples. The discovery of new clinically relevant allergens and the use of their recombinant analogs, as well as their precision printing onto microarray surfaces, could improve the accuracy of results. Furthermore, rational design and the use of nanostructured substrates, high-performance immobilization matrices, and sensitive optical labels could enable the development of allergen microarrays that quantitatively detect sAbs with high sensitivity.

## Figures and Tables

**Table 1:** Comparison of glass-based substrates with other substrates for microarrays.

Substrate Material	Substrate Structure	Characteristics	Ref.
Glass vs. silicon with a SiO_2_ layer	Silicon slide with a 90 nm SiO_2_ layer	- The silicon surface yields an approximately tenfold higher sIgE and approximately fivefold higher sIgG fluorescence signal intensities than a glass slide. - Spotting can be performed on small, precut silicon elements, which can be assembled in different formats (e.g., they can be placed in a microplate and detected by a plate reader). Surface matrix, MCP2 coating; spot volume, 300 pL; distance between spots, 500 µm.	[33]
	Silicon slide with a SiO_2_ layer of a varying thickness (20–150 nm)	- The fluorescence signal and signal-to-noise ratio are up to 4 and 6.8 times higher, respectively, compared to a glass slide. - SiO_2_ films have a very low autofluorescence. - The subnanometer surface roughness allows obtaining a more ordered and reproducible coating of the substrate with biomolecules. Surface matrix, a copolymer of N,N-dimethylacrylamide, N-acryloyloxysuccinimide, and 3-(trimethoxysilyl)propyl methacrylate (DMA-NAS-MAPS); spot volume, 400 pL.	[20]
	Silicon slide with an 80 nm SiO_2_ layer	- The substrate provides up to a 15-fold higher fluorescence signal compared to a glass slide. - Patients with low sIgE levels not identified using a glass-based allergen microarray in five out of eight cases are reliably identified using the silicon-based microarray. Surface matrix, DMA-NAS-MAPS; spot volume, 400 pL.	[21]
Glass vs. silicon PC	The PC consists of a periodic surface structure (period, 360 nm; height, 40 nm) fabricated in a SiO_2_ layer (thickness, 800 nm) on a silicon substrate and then coated with a thin film of TiO_2_	- The substrate provides at least a 20-fold increase in sensitivity compared to a glass slide. - The substrate ensures a consistently higher fluorescence intensity for most protein spots, whether high- or low-abundance, compared to a glass slide. - The substrate ensures a better SNR than a glass slide and faint spots that are not differentiated from the background on the glass slide are distinguishable on it. Surface matrix, an aminosilanized GAPSII glass slide or an epoxysilanized photonic crystal.	[34]
Glass vs. quartz PC	The PC device consists of a periodic surface structure (period, 400 nm; height, 40 nm) fabricated in a quartz substrate and then coated with a thin film of TiO_2_ (thickness, 160 nm)	- The LOD on the PC slide is 140 times lower compared to the LOD on the glass control. - A 330-fold increase in the signal-to-noise ratio has been demonstrated for the concentration corresponding to - The LOD on an unpatterned glass surface. - The quartz-based PC has a background fluorescence 5 times lower than the glass slide. Surface matrix, poly(Lys, Phe) conjugated with a model dye (Alexa-647); spot radius, ~200 µm; center-to-center distance, 500 µm. Identical intensity profiles as a function of distance for a line of fluorescent image pixels in spots on the glass and PC surfaces.	[35]
Nitrocellulose-coated glass vs. physicochemically modified silicon substrate	Silicon roughening by reactive ion etching and chemical modification by MPTMS	- Silicon exhibits a low fluorescence at any wavelength. - The surface area of glass substrates is up to 10^4^ times lower compared to a nitrocellulose coated glass slide, but the surface area of silicon can easily be modified by reactive ion etching, which is not possible with glass. - The spots on the physicochemically modified silicon substrate are as small as 500 pg/mL to 10 ng/mL, which is comparable with those on a nitrocellulose-coated glass slide; however, due to the background fluorescence signal of the nitrocellulose, no information can be obtained from these apparent spots because the variation in the background fluorescence is larger than the spot intensities.	[36]

PC, photonic crystal; MPTMS, mercaptopropyltrimethoxysilane; LOD, limit of detection; SNR, signal-to-noise ratio.

**Table 2:** Matrices and methods for allergen immobilization on the substrate surface.

Immobilization Method	Matrix	Characteristics
2D adsorption	Polycarbonate, PVDF, NC, etc.	Non-permanent immobilization; background signal may be enhanced
3D adsorption	Hydrogels, nanoparticles, etc.	High allergen content; good preservation of allergen conformation
Covalent bonding	Maleimide, NHS, carboxylic esters, etc.	Irreversible immobilization, but allergen conformation and activity may be altered
Affinity interaction	Glutathione, Ni^2+^-NTA, etc.	Oriented conjugation is possible, but allergen amount is poorly controlled

PVDF, polyvinylidene difluoride; NC, nitrocellulose; NHS, N-hydroxysuccinimide.

**Table 3:** Comparison of different types of allergens to be used in microarrays.

Allergen Type	Advantages	Drawbacks
Recombinant allergens	Possibility of unlimited production; lower cost of obtaining compared to purified native allergens (in some cases); precision control of the amount to be printed on the microarray	Limited number of isolated and characterized allergens; possible problems with activity due to PTM and folding
Purified native allergens	Almost guaranteed biological activity; stable characteristics of stock solutions; precision control of the amount to be printed on the microarray	Difficulties with isolation and purification; limited sources
Allergen extracts	High biological activity (in most cases); possibility of including currently unidentified allergens	Variability of allergen content in different extracts; possible contaminations; limited sources; possible problems with immobilization; reduced number of immobilized allergens and, hence, decreased detection sensitivity.

PTM, post-translational modification.

**Table 4:** Characteristics of the most common label-free detection techniques.

Label-Free Technique	Measurement Principle	Description	Ref.
SPR	Detection of changes in the refractive index of the detection area. When molecules are bound to the metal surface, the refractive index increases, thus changing the angle of incidence.	The limit of detection of a β-lactoglobulin SPR sensor is 0.164 µg/mL.	[115]
SPRi	SPRi is used to monitor changes in the refractive index and shifts of the position of the resonance angle as a result of a surface binding event or mass accumulation. SPRi monitors the variations in reflectivity occurring at a fixed angle (working angle) with time.	To enhance the SPRi signal, magnetic beads coupled with secondary anti-IgE antibodies are used. As a result, IgE detection limits of 0.5–1 pg/mL have been achieved.	[116]
Ellipsometry	The change in the polarization of light reflected from the sample surface is measured from the amplitude ratio of two perpendicularly polarized beams.	Simultaneous quantitative detection of three tumor markers has been performed using an ellipsometry biosensor. A 2–5 ng/mL limit of detection has been achieved.	[117]
Interferometry	The read-out platform employs a label-free format by reading the interferometric signal of each of the BICELLs and measuring the binding events that take place on them.	The improved nitrocellulose-based sensing surface of BICELLs ensures a limit of detection as low as 0.7 kU/L, close to that of ImmunoCAP^®^ (0.35 kU/L).	[118]
Mass spectrometry	A nitrogen laser desorbs the protein/energy-absorbing molecule mixture from the array surface, enabling the detection of the proteins captured by the array by mass spectroscopy methods.	New biomarkers can be discovered in complicated samples. However, the method is not quantitative.	[119]

SPR, surface plasmon resonance; SPRi, surface plasmon resonance imaging; BICELLs, biophotonic sensing cells.

## Data Availability

Not applicable.
